# Eupatilin Ameliorates Lipopolysaccharide-Induced Acute Kidney Injury by Inhibiting Inflammation, Oxidative Stress, and Apoptosis in Mice

**DOI:** 10.3390/cimb45090444

**Published:** 2023-08-23

**Authors:** Kiryeong Kim, Hyo-Lim Hong, Gyun Moo Kim, Jaechan Leem, Hyun Hee Kwon

**Affiliations:** 1 Department of Internal Medicine, School of Medicine, Daegu Catholic University, Daegu 42472, Republic of Korea; kileyoung93@naver.com (K.K.); hlhong@cu.ac.kr (H.-L.H.); 2 Department of Emergency Medicine, School of Medicine, Daegu Catholic University, Daegu 42472, Republic of Korea; emprof@cu.ac.kr; 3 Department of Immunology, School of Medicine, Daegu Catholic University, Daegu 42472, Republic of Korea

**Keywords:** lipopolysaccharide, sepsis, acute kidney injury

## Abstract

Acute kidney injury (AKI) is a common complication of sepsis. Eupatilin (EUP) is a natural flavone with multiple biological activities and has beneficial effects against various inflammatory disorders. However, whether EUP has a favorable effect on septic AKI remains unknown. Here, we examined the effect of EUP on lipopolysaccharide (LPS)-evoked AKI in mice. LPS-evoked renal dysfunction was attenuated by EUP, as reflected by reductions in serum creatinine and blood urea nitrogen levels. LPS injection also induced structural damage such as tubular cell detachment, tubular dilatation, brush border loss of proximal tubules, and upregulation of tubular injury markers. However, EUP significantly ameliorated this structural damage. EUP decreased serum and renal cytokine levels, prevented macrophage infiltration, and inhibited mitogen-activated protein kinase and NF-κB signaling cascades. Lipid peroxidation and DNA oxidation were increased after LPS treatment. However, EUP mitigated LPS-evoked oxidative stress through downregulation of NPDPH oxidase 4 and upregulation of antioxidant enzymes. EUP also inhibited p53-mediated apoptosis in LPS-treated mice. Therefore, these results suggest that EUP ameliorates LPS-evoked AKI through inhibiting inflammation, oxidative stress, and apoptosis.

## 1. Introduction

Acute kidney injury (AKI) is defined by the presence of any of the following: (1) an increase in serum creatinine of 0.3 mg/dL or greater within 48 h; (2) a 1.5-fold or greater increase in serum creatinine from baseline within the past 7 days; or (3) urine volume less than 0.5 mL/kg/h for at least 6 h [1]. Accumulating evidence suggests that AKI is related to a high risk for mortality, progression of chronic kidney disease, and other organ failure [2]. Sepsis is a serious condition characterized by organ dysfunction due to dysregulation of the immune response to infection [3]. The excessive pro-inflammatory process in sepsis contributes to the development of kidney damage [4]. Indeed, sepsis is the most common cause of AKI in critically ill patients, accounting for about 50% of all AKI patients admitted to the intensive care unit [4,5]. Septic AKI is known to be strongly associated with poor clinical outcomes [6]. Standard management of septic AKI includes fluid resuscitation, use of vasopressors, and early antibiotic administration [4,5]. However, these therapeutic strategies for septic AKI are supportive and non-specific. Therefore, discovering new drugs that are effective for septic AKI would be of great clinical significance. Accumulating evidence suggests that anti-inflammatory strategies may be useful in patients with sepsis who are in a hyperinflammatory state [7]. Interestingly, anti-inflammatory therapies have shown significant benefit in the treatment of cytokine storm in severe COVID-19, a sepsis-like illness [7].

A clear understanding of the mechanism of septic AKI is very important for the development of effective therapeutics, but despite many efforts to date, its precise underlying mechanism remains largely unknown. However, according to the results of studies so far, activation of innate immunity through the host’s response to danger-associated molecular patterns (DAMPs) or pathogen-associated molecular patterns (PAMPs) after infection is known to be essential in the pathophysiology of septic AKI [4,6]. DAMPs are endogenous molecules released from dying cells in response to cellular damage [8]. Meanwhile, PAMPs are molecules derived from microorganisms and interact with pattern recognition receptors [9]. Lipopolysaccharide (LPS) is a well-known PAMP located on the cell wall of Gram-negative bacteria [9]. LPS can increase the generation of cytokines and reactive oxygen species (ROS) by binding to Toll-like receptor 4 (TLR4) expressed on the surface of immune cells [4,6]. Activated immune cells infiltrate damaged tissues and amplify tissue injury [4,6]. In addition, oxidative stress can contribute to the development of septic AKI by activating apoptotic pathways in tubular epithelial cells [10].

Flavones are one of the subclasses of flavonoids and are secondary metabolites abundant in plants [11]. Flavones have several biological functions such as antimicrobial, anti-inflammatory, antioxidant, and anti-tumor effects [11]. Thus, they are considered as a promising source for drug development. Eupatilin (EUP) is a bioactive flavone found in a variety of medicinal plants, particularly in the genus *Artemisia* [12]. Previous studies have shown that EUP exerts multiple biological activities including anti-inflammatory, antioxidant, and anti-apoptotic effects [13,14,15]. Accumulating evidence suggests that EUP inhibits excessive production of pro-inflammatory cytokines such as tumor necrosis factor-α (TNF-α) in animal models of gastric mucosal injury [16], inflammatory skin diseases [17,18], asthma [19], anaphylactic shock [20], and colitis [21]. Furthermore, Jeong et al. reported the beneficial effect of EUP on renal injury associated with ischemia/reperfusion in mice [22]. EUP also protected kidney epithelial cells from cisplatin-induced injury [23]. However, whether EUP has a protective role in septic AKI remains unknown. Thus, in this study, we examined the effect of EUP on LPS-evoked AKI and evaluated its anti-inflammatory, antioxidant, and anti-apoptotic activities.

## 2. Materials and Methods

### 2.1. Animal Procedures and Treatments

Male C57BL/6 mice (7 weeks old) were acquired from HyoSung Science (Daegu, Republic of Korea). All animal procedures were approved by the Institutional Animal Care and Use Committee of the Daegu Catholic University Medical Center (DCIAFCR-221007-26-Y). The mice were housed under standard temperature (20–24 °C) and humidity (60~70%) conditions. The mice were arbitrarily allocated to four groups (*n* = 8 in each group): the control group (Con), the EUP group, the LPS group, and the LPS+EUP group. The LPS and LPS+EUP groups received a single intraperitoneal administration of LPS (10 mg/kg; Sigma-Aldrich, St. Louis, MO, USA). EUP was obtained from Sigma-Aldrich and dissolved in 5% hydroxypropyl methylcellulose. In the EUP and the LPS+EUP groups, EUP (10 mg/kg) was intragastrically administered 1 h after LPS treatment. The Con and LPS groups were given an equal volume of the vehicle. The doses of EUP and LPS were chosen based on previous literature [22,24]. All mice were sacrificed 24 h after LPS treatment. Blood and kidney tissues were immediately harvested.

### 2.2. Biochemical Analysis of Serum and Renal Tissue

Serum creatinine and blood urea nitrogen (BUN) levels were measured using a biochemical analyzer (Hitachi, Osaka, Japan). Serum TNF-α, interleukin-6 (IL-6), and IL-1β levels were assessed using ELISA kits (R&D Systems, Minneapolis, MN, USA). Renal malondialdehyde (MDA) levels were measured using an MDA assay kit (Sigma-Aldrich, St. Louis, MO, USA).

### 2.3. Histological Analysis and Immunohistochemical (IHC) Staining

The kidneys were fixed and embedded in paraffin. The blocks were sectioned and stained with periodic acid-Schiff (PAS). Tubular injury was assessed in 5 random fields (400×) per sample. The percentage of damaged area was evaluated and scored by using a semiquantitative scale: 0, 0%; 1, ≤10%; 2, 11–25%; 3, 26–45%; 4, 46–75%; and 5, 76–100% [25]. The sections were also immunostained with antibodies against neutrophil gelatinase-associated lipocalin (NGAL; Santa Cruz Biotechnology, Santa Cruz, CA, USA), F4/80 (Santa Cruz Biotechnology, Santa Cruz, CA, USA), and 4-hydroxynonenal (4-HNE; Abcam, Cambridge, MA, USA). The percentage of area stained with NGAL or 4-HNE was analyzed by examining five random fields (400×) per sample using the i-Solution DT software version 11.0 (IMT i-Solution, Coquitlam, BC, Canada). The number of F4/80-stained cells was counted in 10 random fields (400×) per sample.

### 2.4. Immunofluorescence (IF) Staining

To identify brush border loss in the proximal tubule, the kidney sections were incubated with an FITC-conjugated lotus tetragonolobus lectin (LTL; Vector Laboratories, Burlingame, CA, USA) [26]. The percentage of positive staining for LTL was evaluated in 10 random fields (400×) per sample. To evaluate oxidative DNA damage, the sections were probed with an antibody against 8-hydroxy-2′-deoxyguanosine (8-OHdG; Santa Cruz Biotechnology, Santa Cruz, CA, USA) [27]. Nuclei were stained DAPI. The number of 8-OHdG-stained cells were counted in 10 random fields (1000×) per sample.

### 2.5. Western Blotting

Total proteins were extracted from kidneys using a lysis buffer. Extracted proteins were separated on gradient sodium dodecyl sulfate polyacrylamide gels and transferred to nitrocellulose membranes. The membranes were probed with primary antibodies against NGAL (Santa Cruz Biotechnology, Santa Cruz, CA, USA), kidney injury molecule-1 (KIM-1; Abcam, Cambridge, MA, USA), c-Jun N-terminal kinase (JNK; Cell Signaling Technology, Danvers, MA, USA), p-JNK (Cell Signaling Technology, Danvers, MA, USA), extracellular signal-regulated kinase (ERK; Cell Signaling Technology, Danvers, MA, USA), p-ERK (Cell Signaling Technology, Danvers, MA, USA), p38 (Cell Signaling Technology, Danvers, MA, USA), p-p38 (Cell Signaling Technology, Danvers, MA, USA), NF-κB p65 (Cell Signaling Technology, Danvers, MA, USA), p-NF-κB p65 (Cell Signaling Technology, Danvers, MA, USA), NADPH oxidase 4 (NOX4; Novus Biologicals, Littleton, CO, USA), manganese superoxide dismutase (MnSOD; Abcam, Cambridge, MA, USA), p53 (Cell Signaling Technology, Danvers, MA, USA), Bax (Santa Cruz Biotechnology, Santa Cruz, CA, USA), and glyceraldehyde-3-phosphate dehydrogenase (GAPDH; Cell Signaling Technology, Danvers, MA, USA). The signals were detected using chemiluminescence detection reagents (Thermo Fisher Scientific, Waltham, MA, USA).

### 2.6. Quantitative Real-Time RT-PCR

Total RNA was extracted from kidneys using the TRIzol method and was reverse-transcribed into the cDNA using the PrimeScript RT Reagent Kit (TaKaRa, Tokyo, Japan). The qRT-PCR was performed according to the manufacturer’s instruction (Thermal Cycler Dice Real Time System III; TaKaRa, Tokyo, Japan) using primers (Table 1) and the Power SYBR Green PCR Master Mix (Thermo Fisher Scientific, Waltham, MA, USA). Data were analyzed using the 2^−ΔΔCT^ method and GAPDH was used as an internal control.

### 2.7. TUNEL Assay

Assay was performed using a TUNEL assay kit (Roche Diagnostics, Indianapolis, IN, USA) following the manufacturer′s instruction. Positive cells were counted in 10 random fields (600×) per sample.

### 2.8. Statistical Analysis

Data were expressed as the mean ± SEM. As the data except for the tubular injury score followed a Gaussian distribution when evaluated with the Kolmogorov–Smirnov test, statistical differences between the groups were analyzed using the one-way ANOVA analysis with the Bonferroni’s test. The Mann–Whitney nonparametric U test was used to analyze tubular injury scores. *p* < 0.05 was considered statistically significant.

## 3. Results

### 3.1. EUP Attenuated Renal Dysfunction and Tubualr Injury in LPS-Treated Mice

**Figure 1:**
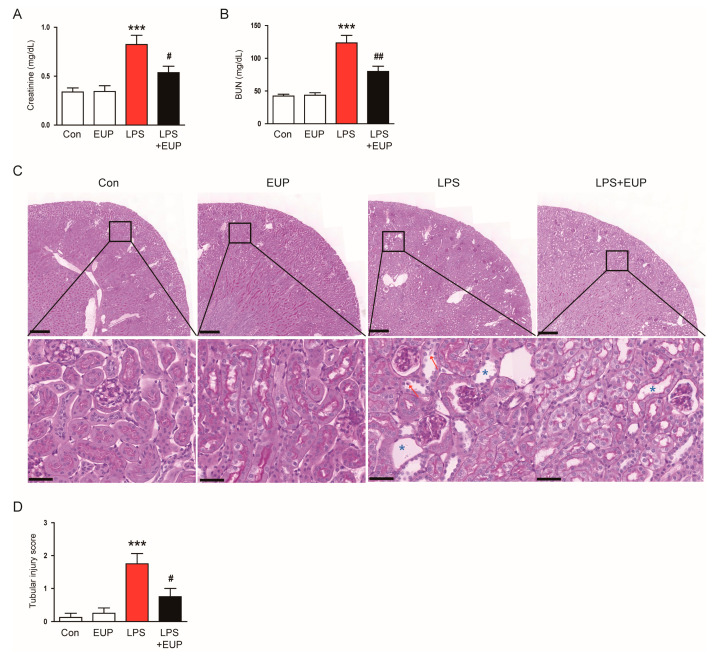
EUP attenuated renal dysfunction and histopathological alterations. (**A**) Serum creatinine levels. (**B**) BUN levels. (**C**) PAS staining. Red arrows indicate tubular cell detachment. Blue asterisks indicate tubular dilatation. Scale bars: 250 μm (upper panel) and 40 μm (lower panel). (**D**) Tubular injury score. *n* = 8 per group. *** *p* < 0.001 vs. Con. ^#^ *p* < 0.05 and ^##^ *p* < 0.01 vs. LPS.

To investigate the action of EUP on LPS-evoked AKI, we first measured serum creatinine and BUN levels, which are renal function indicators [28,29]. LPS injection increased serum creatinine and BUN levels in mice, whereas EUP significantly mitigated LPS-evoked renal dysfunction (Figure 1A,B). PAS staining of kidney sections showed histopathological abnormalities such as tubular cell detachment and tubular dilatation after LPS injection, and EUP attenuated this tubular structural damage in the kidney (Figure 1C,D). On the other hand, administration of EUP alone did not significantly affect renal function and structure (Figure 1A–D).

**Figure 2:**
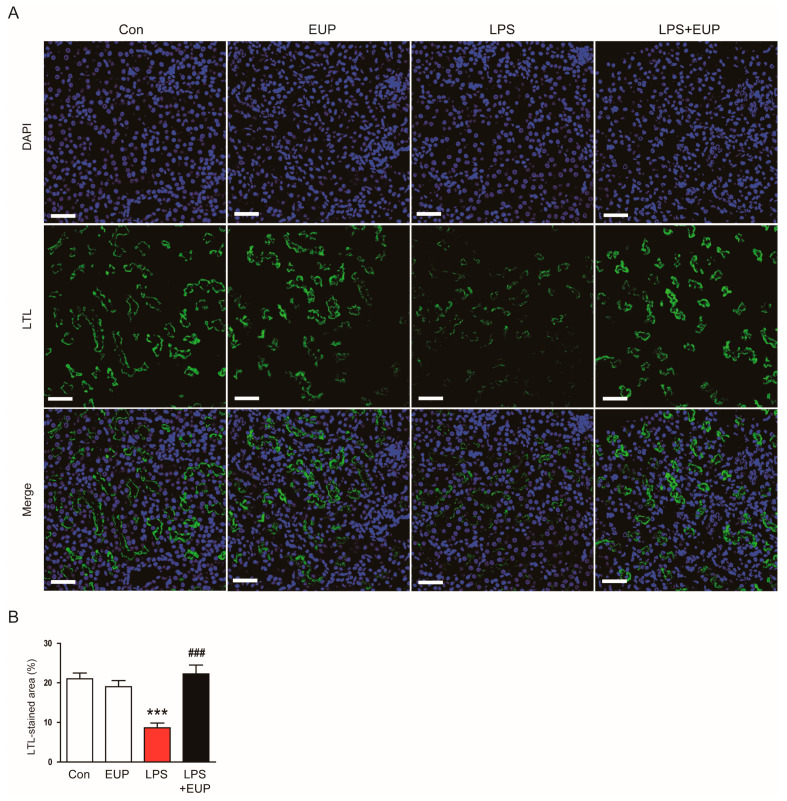
EUP attenuated brush border loss in the proximal tubule. (**A**) LTL staining (green). Scale bar: 50 μm. (**B**) Percentage of the LTL-stained area per field. *n* = 8 per group. *** *p* < 0.001 vs. Con. ^###^ *p* < 0.001 vs. LPS.

IF staining with an FITC-conjugated LTL also showed that LPS treatment resulted in brush border loss in the proximal tubule; however, these changes were reversed by EUP (Figure 2A,B).

**Figure 3:**
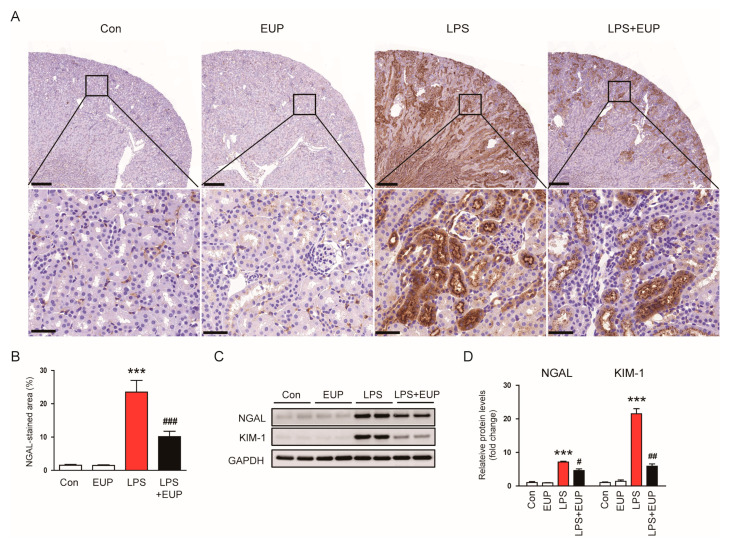
EUP decreased renal expression of NGAL and KIM-1. (**A**) IHC staining for NGAL. Scale bars: 250 μm (upper panel) and 40 μm (lower panel). (**B**) Percentage of the NGAL-stained area per field. (**C**) Western blotting of NGAL and KIM-1. (**D**) Relative band densities of NGAL and KIM-1. *n* = 8 per group. *** *p* < 0.001 vs. Con. ^#^ *p* < 0.05, ^##^ *p* < 0.01 and ^###^ *p* < 0.001 vs. LPS.

To confirm the action of EUP on renal tubular injury, we stained kidneys with an anti-NGAL antibody. NGAL is widely used as renal tubular injury marker because it is produced in renal tubular epithelial cells and increases rapidly after tubular injury [30,31]. The percentage of NGAL-stained area increased after LPS injection, whereas EUP decreased renal NGAL expression in LPS-treated mice (Figure 3A,B). This result was confirmed by Western blotting (Figure 3C,D). Renal expression of KIM-1, another renal tubular injury marker [28], was also increased after LPS injection, and EUP significantly decreased renal KIM-1 protein expression (Figure 3C,D).

### 3.2. EUP Alleviated LPS-Induced Inflammatory Responses

**Figure 4:**
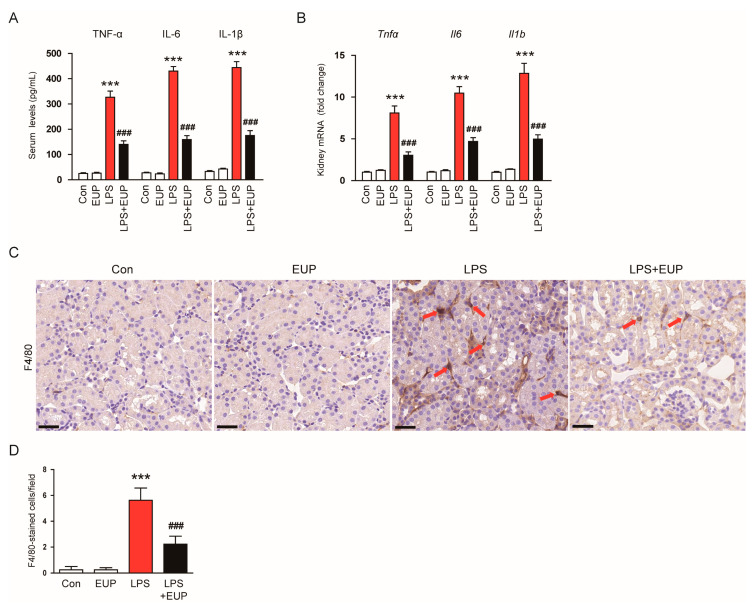
EUP alleviated inflammatory responses. (**A**) Serum levels of TNF-α, IL-6, and IL-1β. (**B**) Renal mRNA levels of TNF-α, IL-6, and IL-1β. (**C**) IHC staining for F4/80. Scale bar: 30 μm. (**D**) Number of F4/80-positive cells per field. *n* = 8 per group. *** *p* < 0.001 vs. Con. ^###^ *p* < 0.001 vs. LPS.

In septic AKI, an excessive inflammatory response is a major cause of organ dysfunction [4,6]. To assess the action of EUP on inflammatory responses, we measured serum cytokine levels in each group. LPS treatment increased serum levels of TNF-α, IL-6, and IL-1β in mice, whereas EUP mitigated these changes (Figure 4A). EUP also decreased renal mRNA expression of the cytokines (Figure 4B). Macrophage infiltration into kidney tissues plays a key role in renal injury and inflammation [32]. We next conducted IHC staining of kidney sections with an antibody against F4/80, a macrophage marker [33], to evaluate macrophage infiltration. We observed that LPS treatment increased the number of F4/80-stained cells in the kidney (Figure 4C,D). However, EUP significantly inhibited macrophage infiltration (Figure 4C,D).

**Figure 5:**
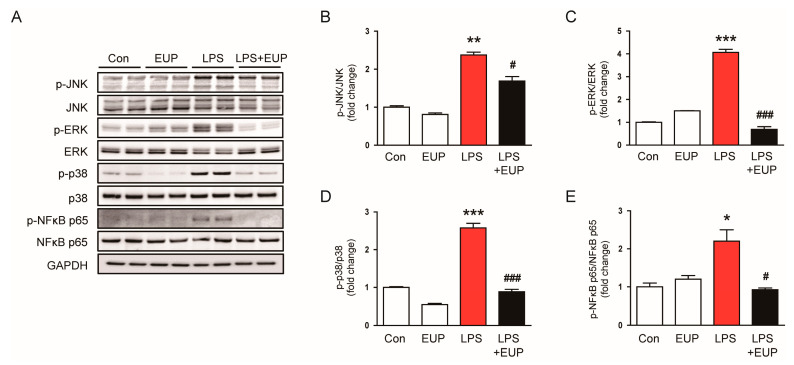
EUP inhibited MAPK-NFκB signaling pathways. (**A**) Western blotting of p-JNK, p-ERK, p-p38, and p-NF-κB p65. (**B**) Relative band density of p-JNK. (**C**) Relative band density of p-ERK. (**D**) Relative band density of p-p38. (**E**) Relative band density of p-NF-κB p65. *n* = 8 per group. * *p* < 0.05, ** *p* < 0.01 and *** *p* < 0.001 vs. Con. ^#^ *p* < 0.05 and ^###^ *p* < 0.001 vs. LPS.

Mitogen-activated protein kinase (MAPK) and NF-κB signaling cascades play key roles in the inflammatory response of septic AKI [34,35,36]. Therefore, we next investigated the action of EUP on MAPK and NF-κB pathways in LPS-treated mice. LPS injection increased the phosphorylated forms of JNK, ERK, p38, and NF-κB p65, and the activation of MAPK and NF-κB cascades were significantly inhibited by EUP (Figure 5A–D).

### 3.3. EUP Mitigated LPS-Induced Oxidative Stress

**Figure 6:**
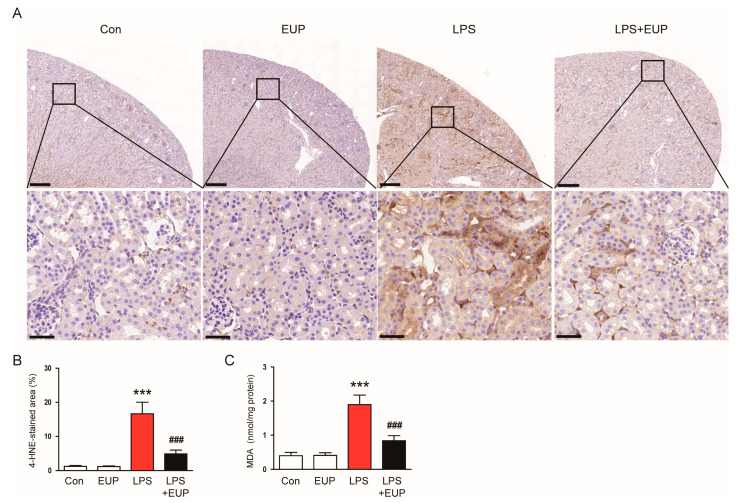
EUP mitigated lipid peroxidation. (**A**) IHC staining for 4-HNE. Scale bars: 250 μm (upper panel) and 40 μm (lower panel). (**B**) Percentage of the 4-HNE-stained area per field. (**C**) Renal MDA levels. *n* = 8 per group. *** *p* < 0.001 vs. Con. ^###^ *p* < 0.001 vs. LPS.

Oxidative stress is a hallmark of sepsis and plays a critical role in the pathophysiology of septic AKI [4,6]. Lipid peroxidation is the oxidative degradation of lipids and is widely used as an indicator of oxidative stress in cells and tissues [37]. Therefore, we performed IHC staining of kidneys with an antibody against 4-HNE, a byproduct of lipid peroxidation [37]. IHC staining revealed that LPS treatment increased 4-HNE expression, but the expression was markedly attenuated by EUP (Figure 6A,B). Renal levels of MDA, another byproduct of lipid peroxidation [38], were also decreased by EUP (Figure 6C).

**Figure 7:**
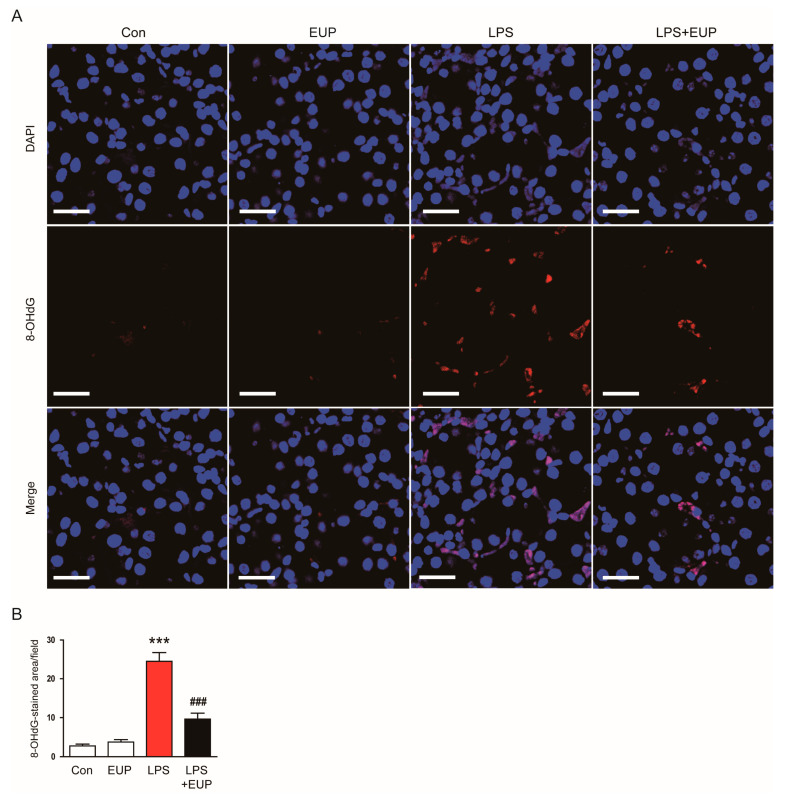
EUP mitigated DNA oxidation. (**A**) IHC staining for 8-OHdG. Scale bar: 30 μm. (**B**) Number of 8-OHdG-stained cells per field. *n* = 8 per group. *** *p* < 0.001 vs. Con. ^###^ *p* < 0.001 vs. LPS.

In addition to lipid peroxidation, we also examined the action of EUP on DNA oxidation. IF staining for 8-OHdG, a DNA oxidation marker [27], revealed that renal 8-OHdG levels increased in kidneys after LPS injection, whereas EUP significantly inhibited DNA oxidation (Figure 7A,B).

**Figure 8:**
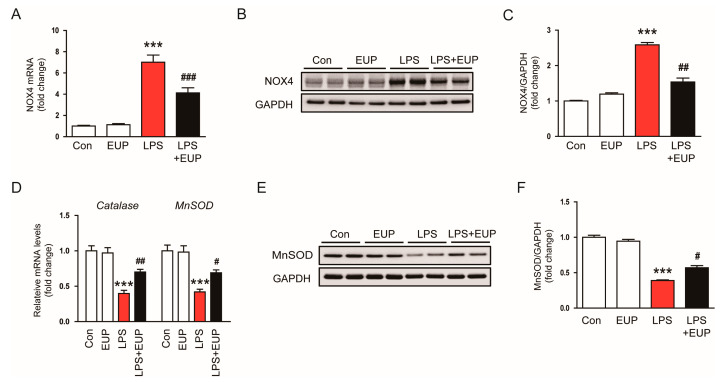
EUP modulated expression of pro-oxidant and antioxidant enzymes. (**A**) Renal mRNA expression of NOX4. (**B**) Western blotting of NOX4. (**C**) Relative band density of NOX4. (**D**) Renal mRNA expression of catalase and MnSOD. (**E**) Western blotting of MnSOD. (**F**) Relative band density of MnSOD. *n* = 8 per group. *** *p* < 0.001 vs. Con. ^#^ *p* < 0.05, ^##^ *p* < 0.01 and ^###^ *p* < 0.001 vs. LPS.

Oxidative stress results from an imbalance between ROS production and antioxidant systems [39]. NOX4 is a primary source of ROS in the kidney and plays an important role in various kidney disorders [40,41,42]. Therefore, we next investigated NOX4 expression in the kidney. We observed an increase in NOX4 mRNA expression in kidneys after LPS injection, and this increase was mitigated by EUP (Figure 8A). This result was confirmed by Western blotting (Figure 8B,C). It has been shown that antioxidant enzymes such as catalase and MnSOD are inhibited in septic AKI [43,44]. The inhibition of catalase and MnSOD resulted in oxidative tissue damage by accumulation of ROS in tissues [43,44]. We also observed that LPS injection reduced mRNA levels of catalase and MnSOD, whereas EUP upregulated their expression in LPS-treated mice (Figure 8D). The increase in MnSOD expression by EUP was confirmed by Western blotting (Figure 8E,F).

### 3.4. EUP Inhibited LPS-Induced Apoptosis

**Figure 9:**
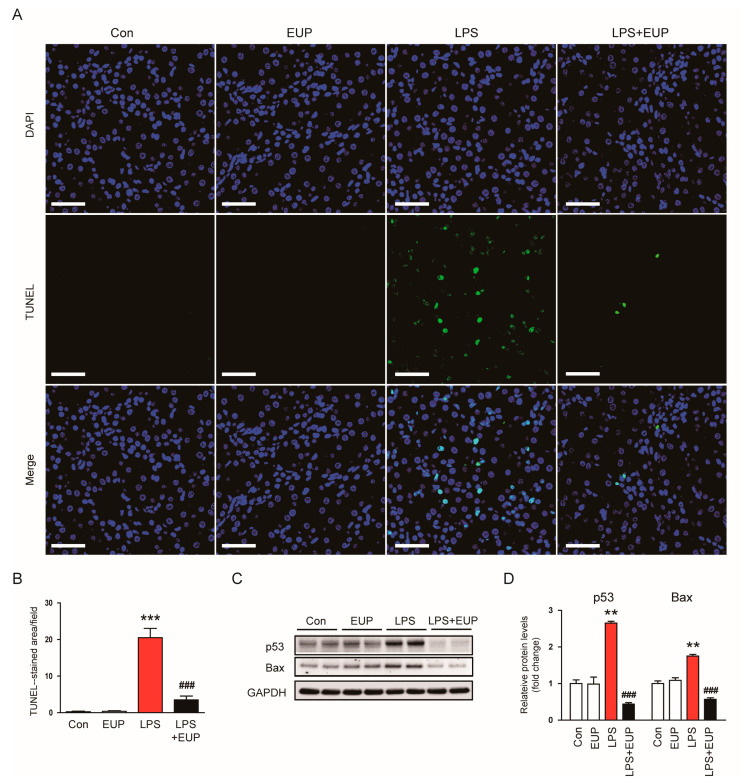
EUP alleviated renal cell apoptosis. (**A**) TUNEL staining (green). Nuclei were stained with DAPI (blue). Scale bar: 50 μm. (**B**) Number of TUNEL-stained cells per field. (**C**) Western blotting of p53 and Bax. (**D**) Relative band densities of p53 and Bax. *n* = 8 per group. ** *p* < 0.01 and *** *p* < 0.001 vs. Con. ^###^ *p* < 0.001 vs. LPS.

Renal cell apoptosis also has important pathogenic implication in septic AKI [4,6]. Therefore, we assessed renal cell apoptosis in kidneys using a TUNEL assay. We observed that LPS treatment increased the number of TUNEL-stained cells in kidneys, and this increase was largely attenuated by EUP (Figure 9A,B). Moreover, protein levels of p53 and Bax increased after LPS injection, whereas EUP markedly decreased their levels (Figure 9C,D).

## 4. Discussion

Septic AKI has a low survival rate and a poor prognosis, placing a significant economic burden on society and patients’ families [6]. However, since complex mechanisms such as inflammation, oxidative stress, and apoptosis are involved in the pathogenesis of sepsis, there are currently no effective drugs that can delay or reverse the development of organ dysfunction. EUP is a flavone found in a variety of medicinal plants, particularly in the genus *Artemisia* and has multiple biological functions including anti-inflammatory, antioxidant, and anti-apoptotic properties [13,14,15]. Therefore, we investigated whether EUP has a favorable effect on septic AKI using a murine model of LPS-evoked AKI.

In the present study, serum creatinine and BUN levels were analyzed in each group of mice to assess the action of EUP on LPS-evoked renal dysfunction. They are well-established indicators of renal function [28,29]. We observed that EUP attenuated LPS-evoked renal dysfunction, as reflected by increases in the levels of both indicators. In septic AKI, damaged renal tubules are known to be characterized by various histopathological abnormalities such as tubular cell detachment, tubular dilatation, and brush border loss [4,6]. In this study, we found that EUP mitigated such histopathological changes in kidneys of LPS-injected mice. EUP also decreased the renal expression of NGAL and KIM-1. NGAL is a member of the lipocalin protein family and its expression is robustly increased in renal tubular epithelial cells following kidney injury [30,31]. KIM-1 is a type 1 transmembrane protein, whose expression is also strongly increased on injured renal tubular epithelial cells [45]. Thus, downregulation of NGAL and KIM-1 indicates amelioration of tubular injury by EUP in LPS-treated mice. Consistent with these results, a previous study demonstrated that EUP attenuated renal ischemia-reperfusion injury in mice, as reflected by reductions in serum creatinine and BUN levels and urinary NGAL and KIM-1 levels [22]. Collectively, these data demonstrated that EUP effectively ameliorated LPS-evoked functional and structural injury in the mouse kidney.

Inflammation has been suggested as a promising target for therapeutic interventions for septic AKI [4,6]. During sepsis, LPS is regarded as a main PAMP and activates TLR4 expressed on the surface of immune cells to stimulate pro-inflammatory cytokine production [4,6]. In this study, we observed that EUP reduced serum and renal levels of cytokines in LPS-treated mice. Previous studies showed that marked macrophage infiltration is observed in the kidneys of mice with septic AKI and that infiltrated macrophages play an important role in the progression of renal injury and inflammation [26,32]. We also found that EUP attenuated the infiltration of F4/80-positive macrophages. These results suggest that EUP exhibits anti-inflammatory action in LPS-induced AKI. To investigate the mechanism underlying the anti-inflammatory effect of EUP, we examined MAPK and NF-κB signaling cascades in the kidney. These pathways are known to play an important role in inflammatory responses [34,35,36]. We found that EUP significantly inhibited activation of MAPK and NF-κB cascades. Similar to our findings, recent studies reported that EUP alleviated septic lung injury by reducing cytokine production and macrophage infiltration [46,47]. EUP inhibited pro-inflammatory cytokine production in LPS-treated macrophages by suppressing the NF-κB pathway [48]. In addition, previous studies reported the suppressive action of EUP on the MAPK/NF-κB pathway in various inflammatory conditions [18,19,20]

Oxidative stress considerably contributes to the development and progression of septic AKI [4,6]. In this study, we observed that the amount of 4-HNE and MDA, which are byproducts of lipid peroxidation, markedly increased in kidneys after LPS injection. However, EUP significantly inhibited lipid peroxidation in LPS-treated mice. DNA oxidation was also inhibited by EUP, as evidenced by a reduction in the number of cells stained with 8-OHdG in kidneys. These results indicate that EUP has an antioxidant effect on LPS-evoked AKI. Consistent with our findings, recent studies showed that EUP ameliorated LPS-induced acute lung injury [47], alcoholic liver disease [49], and osteoarthritis [50] in rodents through inhibiting oxidative stress. In addition, we found that EUP downregulated NOX4 expression and upregulated catalase and MnSOD expression. NOX4 is known to produce ROS and cause oxidative injury in septic AKI [40,41,42]. Zhou et al. also showed the suppressive action of EUP on NOX4 expression in a murine model of dextran sodium sulphate-induced colitis [21]. Catalase and MnSOD are key antioxidant enzymes, and their expression and activity are suppressed in septic AKI [43,44]. Therefore, these data suggest that the regulation of pro-oxidant and antioxidant enzymes by EUP is critically involved in LPS-evoked oxidative stress.

Oxidative stress can also stimulate apoptotic pathways in tubular epithelial cells [51]. In this study, we found that EUP inhibited LPS-evoked apoptosis in kidneys, as reflected by a reduction in the number of TUNEL-stained cells. EUP also decreased protein levels of p53 and Bax. p53 is a transcription factor that regulates the expression of pro-apoptotic proteins such as Bax [52]. During apoptosis, Bax translocates from the cytoplasm to the mitochondrial membrane, resulting in mitochondrial outer membrane permeabilization, caspase activation and apoptotic cell death [53]. Therefore, our findings indicate that EUP inhibits p53-dependent apoptosis in the kidney of LPS-treated mice. Consistent with our findings, the anti-apoptotic activity of EUP has been reported in animal models for various human disorders such as Parkinson’s disease [54], intracerebral hemorrhage [55], and cerebral ischemia [56]. In vitro studies also reported that EUP exerts an anti-apoptotic effect in kidney epithelial cells [23], chondrocytes [57], cardiomyocytes [58], and hepatocytes [59].

Previous studies have shown that EUP activated nuclear factor erythroid 2-related factor 2 (Nrf2) signaling pathways to inhibit inflammation, oxidative stress, and apoptosis in animal models of asthma [19] and cisplatin-induced AKI [60]. Song, et al. reported that EUP protected esophageal epithelial cells from indomethacin-induced cellular injury by activating Nrf2 and upregulating its target gene, heme oxygenase-1 (HO-1) [61]. Nrf2 is a transcription factor that regulates antioxidant enzymes such as HO-1, catalase, and MnSOD [62]. Nrf2 also can inhibit MAPK/NF-κB-mediated inflammatory responses and p53-dependent apoptosis by reducing ROS production [62,63]. Although we did not examine the effect of EUP on Nrf2 in this study, it is possible that EUP increased the expression of antioxidant enzymes, probably through activating the Nrf2 pathway. Further studies will be required to elucidate more detailed mechanisms underlying the protective effect of EUP in septic AKI. Furthermore, EUP has also been shown to exert beneficial effects against other AKI models, such as renal ischemia–reperfusion injury [22] and cisplatin-induced AKI [60]. However, as the potential effect of EUP on chronic kidney disease remains unknown, this will be an interesting topic for future research.

In this study, we used a mouse model of LPS-induced AKI to evaluate the effect of EUP on septic AKI. The LPS mouse model has several advantages, including technical ease and high reproducibility [64]. However, this model is generally more suitable for studying the pathophysiological process of endotoxemia rather than sepsis and may not accurately reflect the characteristic features of human sepsis [64]. Moreover, a variety of clinical situations, such as pre-existing renal injury and persistent pro-inflammatory factors other than sepsis itself, need to be considered in order to translate the results of this study into clinical practice.

**Figure 10:**
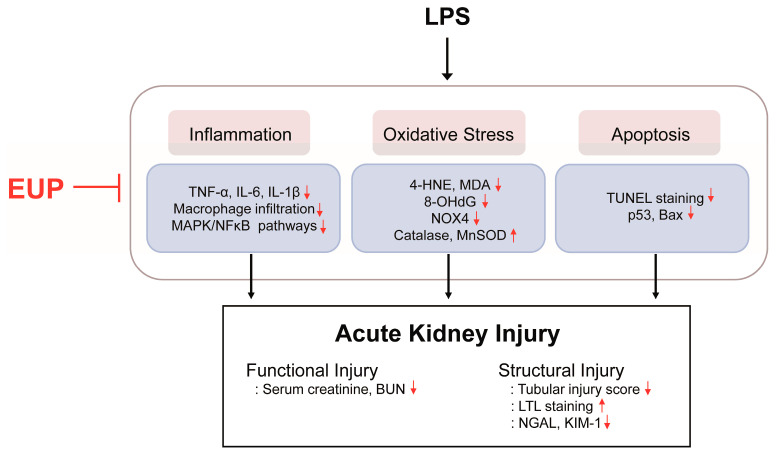
Schematic summary of the results of the present study. EUP ameliorated LPS-induced functional and structural renal injury through inhibiting inflammation, oxidative stress, and apoptosis.

In conclusion, our data demonstrated that EUP has a protective effect on LPS-evoked AKI (Figure 10). EUP effectively attenuates inflammation, oxidative stress, and apoptosis, the key mechanisms of septic AKI, in LPS-treated mice.

## Figures and Tables

**Table 1:** List of primers.

Gene	Primer Sequence (5′→3′)	Accession No.
*Tnfα*	Forward: CCAACGGCATGGATCTCAAAGACA Reverse: AGATAGCAAATCGGCTGACGGTGT	NM_013693
*Il6*	Forward: CCGGAGAGGAGACTTCACAAG Reverse: CAGAATTGCCATTGCACAAC	NM_031168
*Il1* *b*	Forward: TCGCAGCAGCACATCAACAAG Reverse: TCCACGGGAAAGACACAGGTAG	NM_008361
*NOX4*	Forward: CCCTAGCAGGAGAACAAGA Reverse: AACAAGCCACCCGAAAC	NM_015760
*Catalase*	Forward: CACTGACGAGATGGCACACTTTG Reverse: TGGAGAACCGAACGGCAATAGG	NM_009804
*MnSOD*	Forward: GGTCGCTTACAGATTGCT Reverse: CTCCCAGTTGATTACATTCC	NM_013671
*Gapdh*	Forward: CCAGCAAGGACACTGAGCAAGA Reverse: TCCCTAGGCCCCTCCTGTTAT	NM_008084

## Data Availability

Data are included in the article.
